# “Convivência” Groups: Building Active and Healthy Communities of Older Adults in Brazil

**DOI:** 10.1155/2012/612918

**Published:** 2012-07-08

**Authors:** Tânia R. Bertoldo Benedetti, Eleonora d'Orsi, Andiara Schwingel, Wojtek J. Chodzko-Zajko

**Affiliations:** ^1^Department of Physical Education, Center of Sports, Federal University of Santa Catarina, Campus Universitário Reitor João David Ferreria Lima, Caixa Postal 476, Trindade, 88040-900 Florianópolis, SC, Brazil; ^2^Department of Public Health, Center of Health Science, Federal University of Santa Catarina, Campus Universitário Reitor João David Ferreria Lima, Caixa Postal 476, Trindade, 88040-900 Florianópolis, SC, Brazil; ^3^Department of Kinesiology and Community Health, University of Illinois at Urbana-Champaign, 212A Huff Hall MC-588, 1206 South Fourth Street, Champaign, IL 61820, USA; ^4^Department of Kinesiology and Community Health, University of Illinois at Urbana-Champaign, Louise Freer Hall, 906 S. Goodwin Avenue, Urbana, IL 61801, USA

## Abstract

In old age, social groups can be a crucial component for health and well-being. In 2009-2010, a follow-up survey was carried out in Florianópolis, Brazil to understand the impact of a variety of programs established since 2002 that were designed to enhance social activities among the older adult population. This study employed two surveys within the population of older adults in Florianópolis. The first survey interviewed a total of 875 older adults in 2002, and the second survey involved 1,705 older adults between 2009 and 2010. By 2010, many new programs were offered in the community and the enrollment of older adults in social programs followed similar trends. “Convivência” groups stood out as extremely popular social groups among this population. This paper discusses some of the potential outcomes associated with participation in “convivência” groups.

## 1. Introduction

Regular engagement in social activities is an important component of successful aging [1]. Having family and friends to spend time with helps people to find their social identity and purpose in life. Social activity starts with the family at home and goes beyond to schools, work, clubs, community, and faith-oriented groups. These opportunities are very important to people's well-being and quality of life [2, 3].

 Social groups are examples of structured opportunities for people that share similar interests to get together. These interests could range from religion, politics, sports and leisure, and education, among others [4]. Although there is no conclusive evidence of causality, a positive association between social group participation and good health is found in the literature. Social groups are important to people and they play different roles throughout the life course. Older adults engaged in social groups share better physical and mental health than their counterparts who are not engaged [5–9]. Studies show that on average, socially engaged older adults have less depression [6, 10], live more independently [8, 11], have better physical and cognitive functioning [12], have higher levels of life satisfaction [9, 13], and are more likely to be engaged in healthy lifestyles [14–17].

Studies conducted in Brazil have reported that only a small proportion of older adults regularly participate in social groups [18, 19]. Some barriers that prevent older Brazilians from engaging in social groups are meetings held in inconvenient locations, lack of time due to family caregiving roles, and lack of company to motivate regular participation [20].

Efforts to increase social participation among older adults have been initiated worldwide. In the early 1990s, Brazil initiated a program from city halls and faith-based organizations to increase the social engagement of older adults. Public efforts were devoted to the creation of community-based social groups known as “convivência” groups. This initiative was financially supported by the Brazilian National Public Policy for Older Adults in 2003 [21] and was consistent with the strategies identified in the 2002 World Assembly on Ageing held in Madrid, Spain [22].

“Convivência” groups were designed to promote social exchanges among older adults. This is an effort grounded in public health that aims to promote active aging by encouraging healthy lifestyles, independence, productivity, and participation in civic activities [23]. The structure of “convivência” groups involves a daily three-hour period in the afternoon during which a number of different leisure and educational activities are made available to participating older adults aged 60+. Among the most popular activities are folk dance, bingo, seminars about health, exercise classes, choirs, ballroom dance, art craft activities, and watching theater and/or dance performances. Meetings are held in church halls and community centers, and have a participation of 30 to 100 people per day [24, 25].

“Convivência” groups are part of a national program for older adults in Brazil. There have been some studies to document the benefits of this initiative in different parts of the country [26]. In 2002, a comprehensive survey was conducted in southern Brazil which revealed that 20% of older adults were participating regularly in “convivência” groups. The majority of participants were women of low income and with little education [20, 25, 27]. A positive association was found between participating in “convivência” groups and participating in physical activities. Older adults engaged in “convivência” groups were found to be more physically active than their counterparts who were not engaged. Findings of this study were disseminated by the local media and through publications [28]. As a result, the municipality of Florianópolis launched in 2006 a program called “*Capital do Idoso*.” This ongoing program was developed to improve public health in four target areas of intervention: prevention, promotion, therapy, and rehabilitation.

In 2009-2010, a follow-up survey was conducted to understand the impact of programs on social engagement among the older adult population [29]. The changes, benefits, and challenges of these public efforts for the older adult population and for the city as a whole are documented in this paper.

## 2. Methods

This paper was based on two surveys conducted in Florianópolis, capital of Santa Catarina State, Southern Brazil. Florianópolis is a middle size city with approximately 421,203 residents [30]. The first survey was conducted in 2002, and the second in 2010 [28, 29]. The first survey interviewed a total of 875 older adults (437 men and 438 women) with average age of 71.6 ± 7.9 years. In 2002, the population of older adults represented 8.4% of the total population. The data were collected by the research team from September to December 2002. This research was approved by the Ethics Committee for Research on Human Beings of the Universidade Federal de Santa Catarina, Brazil (Protocol no. 051/2001).

The second survey was conducted between 2009 and 2010 with 1,705 older adults (616 men and 1089 women). In 2010, the population of older adults in Florianópolis represented 11% of the total population [30]. The average age of the study participants was 70.7 ± 8.0 years. The data were collected by the research team between September 2009 and June 2010. The research was approved by the Ethics Committee for Research on Human Beings of the Universidade Federal de Santa Catarina, Brazil (Protocol no. 352/2008). A statement of informed consent was obtained from each participant prior the initiation of data collection. Trained interviewers conducted all interviews. The average time taken to conduct each in-home interview was around 60 minutes.

A Brazilian national database was used to select a representative sample of older persons (Instituto Brasileiro de Geografia e Estatística (IBGE)). Both 2002 and 2009 surveys used randomized census tracts. Each census tract has about 300–350 homes. In 2002, the research team interviewed one older adult man and one woman per census tract, whereas the second survey selected census tracts and homes at random.

### 2.1. Research Instruments

A comprehensive questionnaire was administered to each participant. The questionnaire in survey one included demographic information, physical health, the use of medical and dental services, activities of daily living and falls, physical activity levels, social resources, socioeconomic status, mental health, and needs and issues that affect older adults lives. Survey two also included questions about lifestyle, women's health, eating habits, mobility, functional capacity, environmental opportunities for physical activity, and elder abuse. For the purpose of this paper we report only demographic information, socioeconomic status, and social resources.

The International Physical Activity Questionnaire (IPAQ-long form) was used to assess physical activity levels [31]. The IPAQ was developed as a relatively simple self-report instrument that would be available in many languages and which would enable researchers to estimate physical activity levels in different countries and compare these data. A key feature of the IPAQ questionnaire is its ability to provide, in detail, participation estimates for multiple domains of physical activity, including leisure time physical activity, physical activity for transportation, physical activity in the home, and physical activity at work. Although the IPAQ explores physical activity levels in four domains, in 2010 we chose to use data only from two domains, transportation and leisure time physical activity. For the purpose of this paper, the middle level proposed in the original IPAQ was suppressed, following recommendations from previous surveys using IPAQ with older adults in Brazil [32, 33]. Therefore, older adults who carried out moderate or vigorous physical activities within the four domains for 150 minutes per week or over were classified as more active, whereas those who did not reach 150 minutes per week were classified as less active [31].

Analysis of variance was used to examine differences between surveys for the continuous variables. Adjustments for age, sex, education, and income differences were performed using analysis of covariance. Chi-squared tests were used for the analyses of categorical variables among the surveys. All statistical analyses were performed using SPSS 19.0 for Windows (SPSS Inc., Chicago, IL, USA) and statistical significance was set at *P* < 0.05.

## 3. Results

Table 1 shows the distribution of older adults by gender and socioeconomic status of the two surveys conducted in 2002, and in 2010. The average age of participants was not significantly different between the two surveys. In 2002, it was 71.5 years, and 70.6 years in 2010. The first survey included 50% women, whereas in 2010 they comprised 64% of the sample. In both surveys, the great majority of older adults was married and had less than 8 years of education. Household income source was mainly from public retirement pensions. A decline in overall household income was observed between years 2002 and 2010. Households with higher income levels decreased from 20% in 2002 to 10% in 2010. Illiterate older adults were the single largest socioeconomic group.

Table 2 shows participation of the older adults in “convivência groups”, between 2002 and 2010.

Table 3 shows a list of public programs available to all older adults in Florianópolis city, between years 2002 and 2010. Data collected in 2002 has been published elsewhere [34]. Data from both surveys shows a clear increase in opportunities for social activities among older adults in Florianópolis from 2002 to 2010. By 2010, many new programs that were offered to the community and the enrollment of older adults had also increased. For instance, between 2002 and 2010, the total increase in “convivência” group members was 6,849 users. This outnumbers the 5,972 new users for the all other non-“convivência” physical activity, education, and dance groups offered in the city. “Convivência” groups are viewed as extremely popular social groups among older adults in Florianópolis. Adding totals from Table 3, there were 14,849 reported users of the “convivência” groups in 2010, and 9,388 enrolled in other non-“convivência” programs.

The diversity of types of programs also increased. By 2010, activities such as Pilates, “*bailes*” (ballroom dance), and other physical activities had become very popular. In addition, in the 2010 survey, our data suggest that greater attention was paid by health professionals to the promotion of healthy lifestyles and disease management.

**Figure 1:**
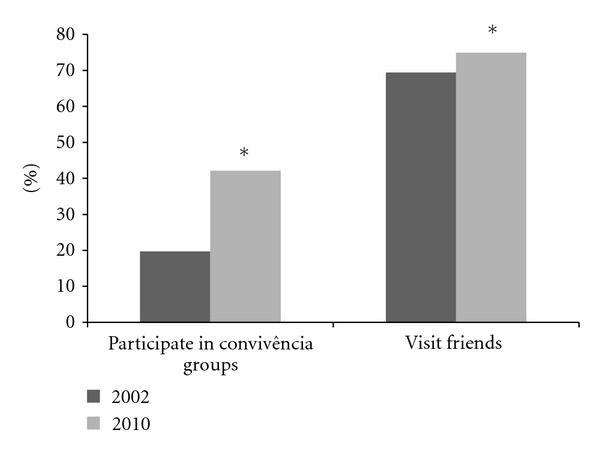
Participation in “convivência” groups and visits to friends between 2002 and 2010.

In order to understand the influence of engagement in social programs, we examined changes in social activity over the time course of the study (Figure 1). Data from year 2010 show a significant increase in participation in “convivência” groups when compared with year 2002; reflecting a greater percentage of older adults engaged in social activities with friends. One of the main goals of the “convivência” groups is to engage local residents of same cohort in social activities and therefore increase and strengthen their circle of friends.

To increase our understanding about lifestyle and healthy behaviors of older adults in Florianópolis, we obtained information on physical activity from older adults participating in “convivência*”* groups.  Table 4 shows the average time spent in physical activity in leisure activities, transportation, and total physical activity in 2002, 2010. By 2010, the time spent in physical activity in transportation has increased significantly. These findings suggest that participating in “convivência” groups had a positive impact on physical activity lifestyle.

## 4. Discussion

In 2002, about 20% of older adults participated in “convivência” groups, whereas by 2010 the participation increased to 42%. By 2010, many more older adults were engaged in social activity with friends and many more were active in physical activity for leisure and transportation when compared to the 2002 survey. This study underscores the potential of “convivência” groups to promote socialization as well as healthy behaviors among the older adults population.

In Brazil, a number of public policy initiatives have facilitated the creation of community-based social groups. In 2003, the Brazilian National Public Policy for Older Adults [21] was implemented, and the Capital do Idoso—Idoso em Forma em Florianópolis program was started in 2006. The Capital do Idoso program was awarded a national recognition [35] for health and prevention services offered to the older adult population. Some researchers have documented the potential association of these public investments with reduced healthcare costs in the municipality. A study reported that selected hospital costs were reduced about 30% between 2006 and 2010 [36]. Other health indicators followed similar trends, such as improvements in sleeping quality, increased self-steem, and decreased usage of the public health system [37].

Our findings suggest that older adults participating in “convivência” groups visited friends more often in 2010 than they did in 2002. In 2010, they were more engaged in other social groups available in the community. Also, in 2010 they spent more time in physical activity during leisure time and for transportation when compared to 2002. Our data suggest that participation in “convivência” groups may have played a role in facilitating positive health behaviors among group members.

A study conducted with older women in Canada examined the influence that social opportunities have on health and functioning capacity [38]. The results of this study showed that as the older women felt more accepted, they presented with less prevalence of diseases; in addition, they had enhanced functional capacity to perform household chores and other daily activities.

The demographic characteristics of the participants in his study are broadly consistent with profile of older Brazilians as a whole.

The aging process is often accompanied by declines in opportunities for social interactions and for establishing new relationships [40]. Retirement from working activities, children leaving home, and loss of loved ones are associated with increased social isolation among older adults [41]. The initial purpose of the Capital do Idoso program was not to promote social integration among the underserved older population of the city, but rather to develop educational activities that target disease prevention, health promotion, therapy, and rehabilitation. Shortly after its start, it became apparent that social engagement of older adults was one of the program's most successful outcomes. It was apparent how socially engaged participants became, and how much more frequently they would leave home to visit their friends. The programs also stimulated an increase in physical activity during transportation and leisure activities. Older adults are the most vulnerable age group for physical inactivity, and they are the least likely to meet physical activity guidelines [42].

The present study has certain limitations that need to be taken into account when considering the study and its contributions. The most important limitation lies in the fact that we did not include control groups, which would have assisted with the interpretation of findings, such as the participation in convivência groups and the positive impact of these groups on health behaviors. For example, it is not clear whether participants in convivência groups differed in their physical activity levels from the general population. In addition, our results may have been influenced by other aspects that were out of our study scope and control, such as increase of regional or national public health campaigns and resources.

In summary, our study suggests that the establishment of convivência groups made a difference to the lives of older adults in Florianópolis. Participating in these groups helped them to be socially engaged and to live actively. Most cities in Brazil have established some kind of convivência group. With this in mind, we can build a new culture of healthy and active aging throughout Brazil, as proposed by the WHO in 2002 [43].

## Figures and Tables

**Table 1:** Characteristics of the older adults living in Florianópolis, Brazil, in years 2002 and 2010.

	2002			2010		
	Overall	Male	Female	Overall	Male	Female
	(*N* = 875)	(*N* = 437)	(*N* = 438)	(*N* = 1705)	(*N* = 616)	(*N* = 1089)
Age, years, mean (SD^∗^)	71.5 (7.9)	71.4 (7.6)	71.7 (8.2)	70.7 (8.0)	70.6 (7.7)	70.9 (8.1)
Marital status, *N* (%)						
Married	536 (61.3)	363 (83.1)	173 (39.5)	993 (58.2)	505 (82)	488 (44.8)
Widow	251 (28.7)	43 (9.8)	208 (47.5)	481 (28.2)	41 (6.7)	440 (40.5)
Divorced	58 (6.6)	22 (5.0)	36 (8.2)	132 (7.7)	51 (8.3)	81 (7.4)
Single	30 (3.4)	9 (2.1)	21 (4.8)	99 (5.8)	19 (3.1)	80 (7.3)
Educational levels, *N* (%)						
None	175 (20.0)	80 (18.3)	95 (21.7)	161 (9.5)	50 (8.2)	111 (10.3)
1 to 8 years	476 (54.0)	215 (49.2)	261 (59.6)	767 (45.3)	256 (41.8)	511 (47.3)
More than 8 years	224 (25.6)	142 (32.5)	82 (18.7)	765 (45.2)	306 (50)	459 (42.5)
Socioeconomic levels (MMW^∗∗^), *N* (%)						
Up to 2	398 (46.4)	142 (32.6)	256 (58.4)	1097 (64.3)	374 (60.7)	723 (66.4)
2 to 6	211 (24.6)	115 (26.4)	96 (21.9)	431 (25.3)	166 (26.9)	265 (24.3)
More than 6	248 (28.9)	167 (38.4)	81 (18.5)	177 (10.4)	76 (12.3)	101 (9.3)

^∗^SD: Standard Deviation; ^∗∗^MMW: Brazilian monthly minimum wage.

**Table 2:** Participation of the older adults in “convivência groups”, 2002 and 2010 in Florianópolis, Brazil.

	Participate in “convivência” groups 2002 (*N* = 875)				Participate in “convivência” groups 2010 (*N* = 1705)			
	No	Yes	*χ* ^2^	*P* value	No	Yes	*χ* ^2^	*P* value
Gender *N* (%)								
Male	387 (55.1)	50 (29.1)	37524.0	<0.001	409 (41.4)	207 (28.8)	28.635	<0.001
Female	315 (44.9)	122 (70.9)			578 (58.6)	511 (71.2)		
Age *N* (%)								
60–69	323 (46)	79 (45.9)	1337.0	0.512	525 (53.2)	411 (57.2)	9600	0.008
70–79	264 (37.6)	59 (34.3)			323 (32.7)	241 (33.6)		
>80	115 (16.4)	34 (19.8)			139 (14.1)	66 (9.2)		
Marital status, *N* (%)								
Married	451 (64.2)	84 (48.8)	20.9	<0.001	592 (60)	401 (55.8)	7.267	0.064
Widow	23 (3.3)	7 (4.1)			60 (6.1)	39 (5.4)		
Divorced	50 (7.1)	8 (4.7)			81 (8.2)	51 (7.1)		
Single	178 (25.4)	73 (42.4)			254 (25.7)	227 (31.6)		
Educational levels, *N* (%)								
None	145 (20.7)	30 (17.4)	0.91	0.637	96 (9.8)	65 (9.1)	0.338	0.845
1 to 8 years	378 (53.8)	97 (56.4)			439 (44.8)	328 (45.9)		
More than 8 years	179 (25.5)	45 (26.2)			444 (45.4)	321 (45)		
Socio-economic levels (in MMW^∗^), *N* (%)^#^								
Up to 2	317 (45.3)	80 (46.5)	0.72	0.868	643 (65.1)	454 (63.2)	0.777	0.678
2 to 6	168 (24)	43 (25)			242 (24.5)	189 (26.3)		
More than 6	201 (28.7)	47 (27.3)			102 (10.3)	75 (10.4)		
Physical activity level, *N* (%)								
Less active	501 (71.4)	118 (68.6)	0.51	0.266	530 (53.7)	330 (46)	9.953	0.002
More active	201 (28.6)	54 (31.4)			457 (46.3)	388 (54)		

*MMW: Brazilian monthly minimum wage; ^#^16 participants did not answer this question.

**Table 3:** Public programs available to older adults in Florianópolis, Brazil, between years 2002 and 2010.

Entities and programs	2002		2010	
	Number of groups	Number of older adults enrolled	Number of groups	Number of older adults enrolled
Municipality programs				
“Convivência"	93	3,500	105	4,509
Exercise and physical activity	57	1,670	111	4,311
Healthy lifestyle and disease management	2	50	94	1,250
University outreach programs				
Exercise and physical activity^∗^	29	805	38	822
Senior education	22	596	45	997
Chambers of commerce (SESC)programs				
“Convivência" and senior social events	23	4,500	26	10,340
Exercise and physical activity	4	200	4	95
Senior education	1	20	13	374
Other programs				
Dancing schools for seniors	0	0	13	81
“Bailes” (dance clubs for seniors)	2	120	12	1,458

^∗^Work Out Groups, Water Aerobics Groups, Swimming Groups, Dance Groups, Physical Activity to Parkinson Disease Groups, Sports Groups; Yoga Groups; Pilates Groups; Walking Groups; Bodybuilding Groups.

**Table 4:** Time spent in physical activity (leisure transportation and total) among older adults participating in “*convivência*” groups, in 2002 and 2010.

	2002			2010				
	(*N* = 875)			(*N* = 1705)			*χ* ^2^	*P* value
Participate in senior social groups, *N* (%)	172 (19.7)			718	42.1		128.65	<0.001
Visit relatives, *N* (%)	637 (72.8)			1194	71.2		0.77	N.S.
Visit friends	607 (69.4)			1256	74.9		8.76	0.003
Overall physical activity (IPAQ)								
Transportation PA, min/wk, mean (SE), 95% CI	48.3	8.1	32.3 to 64.2	120.8	4.7	1.5 to 130.1		<0.001
Transportation PA^∗^, min/wk, mean (SE), 95% CI	46.7	7.7	31.5 to 61.9	125.8	5.5	5.1 to 136.4		<0.001
Leisure PA, mean (SE), 95% CI	108.1	7.1	94.1 to 122.1	128.7	5.2	8.5 to 138.8		0.004
Leisure PA^∗^, mean (SE), 95% CI	113.0	7.8	97.8 to 128.2	130.2	5.5	9.5 to 141.0		0.015

*Adjusted by age, sex, BMI, education, and income.
